# Complete Genome Sequences of 12 Species of Stable Defined Moderately Diverse Mouse Microbiota 2 

**DOI:** 10.1128/genomeA.00951-16

**Published:** 2016-09-15

**Authors:** Yasuhiro Uchimura, Madeleine Wyss, Sandrine Brugiroux, Julien P. Limenitakis, Bärbel Stecher, Kathy D. McCoy, Andrew J. Macpherson

**Affiliations:** a Maurice Müller Laboratories (DKF), Universitätsklinik für Viszerale Chirurgie und Medizin Inselspital, University of Bern, Bern, Switzerland; b Max-von-Pettenkofer Institute and German Centre for Infection Research (DZIF), Munich, Germany

## Abstract

We report here the complete genome sequences of 12 bacterial species of stable defined moderately diverse mouse microbiota 2 (sDMDMm2) used to colonize germ-free mice with defined microbes. Whole-genome sequencing of these species was performed using the PacBio sequencing platform yielding circularized genome sequences of all 12 species.

## GENOME ANNOUNCEMENT

Isobiotic animal models with a defined stable composition are being developed to study host-microbial mutualism under reproducible conditions (1). The commonly used altered Schaedler flora (ASF) of eight species was devised to minimize immunopathology from blooms of nosocomial immigrant microbes (2, 3). Unfortunately, since ASF-colonized mice neither fully recapitulate immune adaptations nor disease models seen in diversely colonized mice, the microbiota is insufficient to model most aspects of host microbial mutualism (1).

Here, we report the genomic sequences of 12 bacterial organisms in a stable defined moderately diverse microbiota in the specific-pathogen-free mouse 2 (sDMDMm2) (4) covering five major phyla of prokaryotes (Table 1). This sDMDMm2 (also termed Oligo-MM^12^) gnotobiotic mouse model has been maintained successfully for over five generations in the clean mouse facility of the University of Bern and LMU Munich. These bacterial strains are publically available from the German Type Culture Collection (DSMZ) (Table 1).

**TABLE 1:** Accession numbers and genome characteristics of sDMDMm2 species

Organism	Total size (bp)	G+C content (%)	No. of genes	DSM no.	Accession no.
*Lachnoclostridium* sp. YL32	7,225,343	48.1	7,759	DSM 26114	CP015399
*Ruminiclostridium* sp. KB18	3,802,817	54.6	3,981	DSM 26090	CP015400
*Bacteroides* sp. I48	4,839,918	42.6	4,262	DSM 26085	CP015401
*Parabacteroides* sp. YL27	3,306,456	50.1	2,790	DSM 28989	CP015402
*Burkholderiales* bacterium YL45	2,923,068	44.1	2,778	DSM 26109	CP015403
*Erysipelotrichaceae* bacterium I46	4,468,295	43.2	4,659	DSM 26113	CP015404
*Blautia* sp. YL58	5,128,792	45.7	5,232	DSM 26115	CP015405
*Flavonifractor plautii* YL31	3,818,500	60.9	3,929	DSM 26117	CP015406
*Bifidobacterium animalis* subsp. *animalis* YL2	1,800,480	60.1	1,555	DSM 26074	CP015407
*Lactobacillus reuteri* I49	2,044,770	38.8	1,988	DSM 32035	CP015408
*Akkermansia muciniphila* YL44	2,745,273	55.7	2,746	DSM 26127	CP015409
*Enterococcus faecalis* KB1	3,026,016	37.2	2,938	DSM 32036	CP015410

Full-genome sequencing of these sDMDMm2 species was carried out from a single colony of each bacterial species grown in brain heart infusion (BHI) medium (Oxoid) supplemented with four chemicals (0.025% cysteine, 0.025% Na_2_S, 0.001% hemin, and 0.00005% menadione) in an anaerobic chamber (10% CO_2_, 10% H_2_, 80% N_2_), except for *Akkermansia muciniphila* YL44 (further supplementation of 0.025% mucin to the BHI medium) and *Lactobacillus reuteri* I49 (grown in MRS medium; Oxoid). Genomic DNA was purified by standard phenol-chloroform extraction. Whole-genome sequencing was carried out using PacBio RS II sequencing platform with a 10-kb insert library and XL/C2 chemistry (5). Hierarchical Genome Assembly Process (HGAP) performed high-quality *de novo* assembly using a single PacBio library preparation. HGAP consisted of preassembly, *de novo* assembly with Celera Assembler, HGAP2 assembly, and circularization (6). The assembled sequences were annotated using the RAST server (7).

### Accession number(s).

All 12 assembled sequences have been deposited in DDBJ/ENA/GenBank under the accession numbers as provided in Table 1.
